# Activity of ceftolozane/tazobactam and imipenem/relebactam against clinical isolates of Enterobacterales and *Pseudomonas aeruginosa* collected in Greece and Italy—SMART 2017–2021

**DOI:** 10.1007/s10096-024-04756-4

**Published:** 2024-05-22

**Authors:** James A. Karlowsky, Sibylle H. Lob, Stephen P. Hawser, Nimmi Kothari, Fakhar Siddiqui, Irina Alekseeva, C. Andrew DeRyke, Katherine Young, Mary R. Motyl, Daniel F. Sahm

**Affiliations:** 1 IHMA, 2122 Palmer Drive, Schaumburg, IL 60173 USA; 2 Department of Medical Microbiology and Infectious Diseases, Max Rady College of Medicine, University of Manitoba, Winnipeg, Manitoba Canada; 3 IHMA, Monthey, Switzerland; 4 Merck & Co., Inc., Rahway, NJ USA; 5 MSD, Dubai, United Arab Emirates

**Keywords:** Ceftolozane/tazobactam, Imipenem/relebactam, Enterobacterales, *Pseudomonas aeruginosa*, Southern Europe, SMART

## Abstract

**Purpose:**

The current study evaluated the in vitro activities of ceftolozane/tazobactam (C/T), imipenem/relebactam (IMI/REL), and comparators against recent (2017–2021) clinical isolates of gram-negative bacilli from two countries in southern Europe.

**Methods:**

Nine clinical laboratories (two in Greece; seven in Italy) each collected up to 250 consecutive gram-negative isolates per year from lower respiratory tract, intraabdominal, urinary tract, and bloodstream infection samples. MICs were determined by the CLSI broth microdilution method and interpreted using 2022 EUCAST breakpoints. β-lactamase genes were identified in select β-lactam-nonsusceptible isolate subsets.

**Results:**

C/T inhibited the growth of 85–87% of Enterobacterales and 94–96% of ESBL-positive non-CRE NME (non-*Morganellaceae* Enterobacterales) isolates from both countries. IMI/REL inhibited 95–98% of NME, 100% of ESBL-positive non-CRE NME, and 98–99% of KPC-positive NME isolates from both countries. Country-specific differences in percent susceptible values for C/T, IMI/REL, meropenem, piperacillin/tazobactam, levofloxacin, and amikacin were more pronounced for *Pseudomonas aeruginosa* than Enterobacterales. C/T and IMI/REL both inhibited 84% of *P. aeruginosa* isolates from Greece and 91–92% of isolates from Italy. MBL rates were estimated as 4% of Enterobacterales and 10% of *P. aeruginosa* isolates from Greece compared to 1% of Enterobacterales and 3% of *P. aeruginosa* isolates from Italy. KPC rates among Enterobacterales isolates were similar in both countries (7–8%). OXA-48-like enzymes were only identified in Enterobacterales isolates from Italy (1%) while GES carbapenemase genes were only identified in *P. aeruginosa* isolates from Italy (2%).

**Conclusion:**

We conclude that C/T and IMI/REL may provide viable treatment options for many patients from Greece and Italy.

## Introduction

Enterobacterales and *Pseudomonas aeruginosa* are the most frequent gram-negative pathogens causing serious infections. Resistance to first-line agents used to treat serious gram-negative infections (β-lactams, including carbapenems, and fluoroquinolones) is a global concern and is increasing with time [1–3]. The prevalence of antimicrobial resistance among gram-negative bacilli has been shown to vary by global region and within global regions [1–3]. In Europe, antimicrobial resistance is higher in southern and eastern countries than northern and western countries [3–8]. Structured surveillance initiatives are important for tracking resistance changes locally, regionally, and globally, and support therapeutic and public health policy revision and new drug development. Documenting the utility of newer β-lactam/β-lactamase inhibitor combination agents such as ceftolozane/tazobactam (C/T) and imipenem/relebactam (IMI/REL) in specific countries in global regions with elevated resistance rates to first-line anti-gram-negative therapies (e.g., southern Europe) is universally informative.

C/T is an antipseudomonal cephalosporin/β-lactamase inhibitor combination that is also active against most ESBL-positive Enterobacterales [9]. C/T is approved by the European Medicines Agency (EMA) and the US Food and Drug Administration (FDA) for the treatment of complicated urinary tract infection, complicated intraabdominal infection, and hospital-acquired and ventilator-associated bacterial pneumonia (HAP and VAP). IMI/REL is a combination of imipenem/cilastatin with relebactam, a non-β-lactam diazaabicyclooctane (DBO) inhibitor of class A (ESBL, KPC) and C (AmpC) β-lactamases [4, 9]. IMI/REL is inactive against MBL-, OXA-48-like-, and GES-producing isolates of gram-negative bacilli [4, 9]. IMI/REL is approved by the EMA and the FDA for HAP and VAP, bacteremia associated with HAP and VAP (EMA only), and infections due to aerobic gram-negative bacilli in adults with limited treatment options (e.g., complicated urinary tract infection, complicated intraabdominal infection).

Previous publication of country-specific in vitro susceptibility testing data for C/T and/or IMI/REL against surveillance isolates of gram-negative bacilli causing patient infections in Greece and Italy is very limited [7, 10, 11]. We evaluated the activity of these two agents and relevant comparators against clinical isolates of gram-negative bacilli collected by clinical laboratories in Greece and Italy as part of the Study for Monitoring Antimicrobial Resistance Trends (SMART) global surveillance program from 2017 to 2021.

## Methods

### Bacterial isolates

Two clinical laboratories in Greece and seven clinical laboratories in Italy participated in the SMART global surveillance program from 2017 to 2021. Each laboratory collected isolates from lower respiratory tract infections (100 isolates/year), intraabdominal infections (75 isolates in 2017 and 50 isolates/year in 2018–2021), urinary tract infections (75 isolates in 2017 and 50 isolates/year in 2018–2021), and bloodstream infections (50 isolates/year in 2018–2021 only). All isolates were shipped to a central laboratory (IHMA, Monthey, Switzerland). Species identification was confirmed using matrix-assisted laser desorption ionization-time of flight (MALDI-TOF) mass spectrometry (Bruker Daltonics, Billerica, MA, USA) by the central laboratory. One isolate per patient per species per year is accepted by the SMART global surveillance program.

### Antimicrobial susceptibility testing

MICs were determined by the CLSI reference broth microdilution method [12, 13]. Custom-made dehydrated broth microdilution panels manufactured by TREK Diagnostic Systems (Thermo Fisher Scientific, Oakwood Village, OH, USA) were used in 2017 and broth microdilution panels prepared at IHMA in 2018–2021. MICs were interpreted by 2022 EUCAST breakpoints [14]. EUCAST does not publish breakpoints for IMI/REL for *Morganellaceae* (*Proteus*, *Providencia*, and *Morganella* spp.) [14]. Imipenem has intrinsically low activity against *Morganellaceae* [14] by a mechanism independent of β-lactamase production [13]. Relebactam does not improve the activity of imipenem against *Morganellaceae*. Therefore, we only analyzed IMI/REL susceptibility for non-*Morganellaceae* Enterobacterales (NME) isolates. An ESBL-positive non-CRE (carbapenem-resistant Enterobacterales) phenotype was defined as an isolate of *Escherichia coli*, *Klebsiella oxytoca*, and *Klebsiella pneumoniae* testing with a ceftriaxone MIC ≥2 mg/L and an ertapenem MIC ≤0.5 mg/L.

### β-lactamase gene detection

Isolates meeting the following phenotypic criteria were screened for β-lactamase genes: NME isolates (excluding *Serratia* spp.) testing with imipenem or imipenem/relebactam MIC values of ≥2 mg/L and *P. aeruginosa* isolates testing with imipenem or imipenem/relebactam MIC values of ≥4 mg/L collected in 2017–2021; NME and *Serratia* spp. isolates testing with ertapenem MIC values of ≥1 mg/L collected in 2017–2018 only; isolates of *Serratia* spp. testing with imipenem MIC values of ≥4 mg/L collected in 2017–2018; and Enterobacterales and *P. aeruginosa* isolates testing with ceftolozane/tazobactam MIC values of ≥4 mg/L and ≥8 mg/L, respectively, collected in 2017–2021. Published multiplex PCR assays were used to screen for the following β-lactamase genes: ESBLs (CTX-M, GES, PER, SHV, TEM, VEB); acquired AmpC β-lactamases (ACC, ACT, CMY, DHA, FOX, MIR, MOX) and the chromosomal AmpC intrinsic to *P. aeruginosa* (PDC, *Pseudomonas*-derived cephalosporinase); serine carbapenemases (GES, KPC, OXA-48-like [Enterobacterales], OXA-24-like [*P. aeruginosa*]); and metallo-β-lactamases (MBLs) (GIM, IMP, NDM, SPM, VIM) [15, 16]. All detected genes encoding carbapenemases, ESBLs, and PDC were amplified using gene-flanking primers and sequenced (Sanger). For *P. aeruginosa* collected in 2020 and 2021 only, isolates were characterized by short-read whole-genome sequencing (Illumina Hiseq 2×150 bp reads) to a targeted coverage depth of 100× [17] and analyzed using the CLC Genomics Workbench (Qiagen). The Resfinder database was used to detect β-lactamase genes [18]. Among 5332 collected Enterobacterales and 1094 *P. aeruginosa*, 16 and 8 isolates, respectively, were not available for molecular characterization and were not included in the denominators for carbapenemase rate calculations. Of these, six and four isolates, respectively, would have qualified for molecular characterization. Per SMART protocol for Enterobacterales isolates collected in 2021, a representative sample of approximately 95% of isolates meeting the criteria for molecular characterization was characterized. Accordingly, of the 171 isolates that met the testing criteria and were available for molecular characterization, eight randomly selected isolates were not characterized. Per SMART protocol for *P. aeruginosa* isolates collected in 2020 and 2021, a representative sample of approximately 75% of isolates meeting the criteria for molecular characterization were characterized (26 randomly selected isolates of 107 qualified isolates were not characterized). For each clinical laboratory, the percentage of qualified isolates collected in 2020 and 2021 that was not characterized was considered when calculating estimated carbapenemase rates.

## Results and discussion

C/T inhibited 85–87% of all collected Enterobacterales and 94–96% of ESBL-positive non-CRE NME isolates from Greece and Italy (Table 1). Our current results for C/T agree closely with a single previous report for clinical isolates of Enterobacterales collected between 2012 and 2018 in both Greece (89% susceptible; *n*=1039) and Italy (86%; *n*=3881) [7]. Only meropenem and amikacin showed higher activity (by 2–7 percentage points) than C/T against all Enterobacterales isolates. In the current study, the majority of molecularly characterized C/T-resistant Enterobacterales isolates (72%, 564/785) from both countries carried a serine carbapenemase or MBL gene. KPCs were the most common carbapenemase gene identified in Enterobacterales isolates from both Greece (7.2%) and Italy (7.9%) confirming earlier reports [4, 5]. MBL genes were identified in 4.3% of Enterobacterales isolates from Greece and 1.2% of isolates from Italy. OXA-48-like enzymes were only identified in Enterobacterales isolates from Italy (1.4%) (Table 2).

**Table 1:** Antimicrobial susceptibility testing results for all Enterobacterales and *P. aeruginosa* isolates and isolates with resistant phenotypes and genotypes

Organism/phenotype/country (*n*)	% Susceptible											
	C/T	IMI/REL	IMI^a^	MEM	ETP	FEP^b^	CAZ^b^	CRO	P/T^b^	LVX^b,c^	AMK	CST
Enterobacterales												
All isolates												
Greece (1367)	86.5	NA	88.0	88.7	87.7	79.2	75.2	76.4	79.6	66.1	89.0	74.1
Italy (3965)	84.6	NA	89.8	90.2	87.6	70.9	66.7	67.0	76.2	64.9	91.4	80.7
NME												
Greece (1133)	84.5	95.1	86.4	86.7	85.4	76.4	73.3	73.6	76.3	67.1	87.9	89.4
Italy (3598)	84.5	98.0	89.0	89.2	86.4	70.0	66.6	66.5	74.2	66.2	91.4	88.9
ESBL-positive non-CRE NME^d^												
Greece (108)	96.3	100	100	100	100	10.2	6.5	0	67.6	19.0	91.7	97.2
Italy (576)	94.4	100	100	100	100	7.6	7.3	0	63.7	23.7	92.9	99.3
KPC-positive NME^e^												
Greece (97)	0	99.0	1.0	1.0	0	0	0	0	0	1.3	15.5	58.8
Italy (307)	0	98.4	0.7	1.6	0	0.3	0	0	0	0.7	37.1	71.0
*P. aeruginosa*												
All isolates												
Greece (183)	83.6	84.2	71.6	71.0	NA	77.0	80.3	NA	78.7	69.4	84.2	100
Italy (911)	92.1	91.4	77.3	77.4	NA	80.5	74.9	NA	71.1	76.9	91.4	99.7
MEM-R												
Greece (31)	22.6	16.1	0	0	NA	12.9	22.6	NA	12.9	9.7	29.0	100
Italy (111)	53.2	36.0	0	0	NA	27.9	24.3	NA	12.6	18.0	50.5	99.1
FEP-R												
Greece (42)	31.0	38.1	21.4	19.0	NA	0	19.0	NA	11.9	16.7	35.7	100
Italy (178)	65.2	66.3	37.1	32.6	NA	0	7.3	NA	4.5	36.5	64.6	100
CAZ-R												
Greece (36)	22.2	30.6	19.4	19.4	NA	5.6	0	NA	11.1	11.1	30.6	100
Italy (229)	69.9	71.2	43.7	41.5	NA	27.9	0	NA	8.7	46.3	69.9	100
P/T-R												
Greece (39)	30.8	35.9	15.4	12.8	NA	5.1	17.9	NA	0	12.8	35.9	100
Italy (263)	74.1	73.4	44.9	41.1	NA	35.4	20.5	NA	0	46.4	72.6	99.6

^a^The results provided for Enterobacterales combined % Susceptible, Increased Exposure values for *Morganellaceae* and % Susceptible values for non-*Morganellaceae* Enterobacterales [14]

^b^The % Susceptible results for *P. aeruginosa* used % Susceptible, Increased Exposure MIC breakpoints defined by EUCAST [14]

^c^Levofloxacin was only tested against Enterobacterales isolates from 2018 to 2021

^d^ESBL-positive non-CRE NME isolates were defined as *E. coli*, *K. oxytoca*, and *K. pneumoniae* isolates testing with a ceftriaxone MIC ≥2 mg/L and an ertapenem MIC ≤0.5 mg/L

^e^KPC-positive NME isolates were MBL-negative (i.e., these excluded five isolates from Greece and seven isolates from Italy that co-carried KPC and an MBL)

*C/T*, ceftolozane/tazobactam; *IMI/REL*, imipenem/relebactam; *MEM*, meropenem; *ETP*, ertapenem; *FEP*, cefepime; *CAZ*, ceftazidime; *CRO*, ceftriaxone; *P/T*, piperacillin/tazobactam; *LVX*, levofloxacin; *AMK*, amikacin; *CST*, colistin; *NME*, non-*Morganellaceae* Enterobacterales; *ESBL*, extended-spectrum β-lactamase; *CRE*, carbapenem-resistant Enterobacterales; *R*, resistant; *NA*, not applicable

**Table 2:** Estimated carbapenemase rates among Enterobacterales, NME, and *P. aeruginosa* isolates

Organism/phenotype/country (*n*^a^)	Estimated % of isolates carrying a carbapenemase			
	MBL	KPC^b^	OXA-48-like^b^	GES
Enterobacterales				
All isolates				
Greece (1366)	4.3	7.2	0	0
Italy (3950)	1.2	7.9	1.4	0
NME				
Greece (1132)	4.9	8.6	0	0
Italy (3583)	1.3	8.7	1.6	0
*P. aeruginosa*				
All isolates				
Greece (183)	10.2	0	0	0
Italy (903)	2.7	0	0	1.7

^a^Only isolates that were available for molecular characterization were included in the denominators for percentage calculations

^b^KPC-positive and OXA-48-like-positive isolates of Enterobacterales and NME were MBL-negative (i.e., these excluded five isolates from Greece and seven isolates from Italy that co-carried KPC and an MBL; and the one isolate from Greece and 11 isolates from Italy that co-carried an OXA-48-like enzyme and an MBL)

*MBL*, metallo-β-lactamase

IMI/REL inhibited 95–98% of all NME, 100% of ESBL-positive non-CRE NME, and 98–99% of KPC-positive NME isolates from Greece and Italy (Table 1). IMI/REL was more active than meropenem (87–89%), ertapenem (85–86%), amikacin (88–91%), and colistin (89%) against NME, while percent susceptible values for cefepime, ceftazidime, ceftriaxone, piperacillin/tazobactam, and levofloxacin were <80%. Levofloxacin was the least active agent tested against all Enterobacterales, NME, and ESBL-positive non-CRE NME isolates in both Greece and Italy. Less than 25% of ESBL-positive non-CRE NME isolates were levofloxacin-susceptible and 64–68% of ESBL-positive non-CRE NME isolates were piperacillin/tazobactam-susceptible. MBL genes were identified in 4.9% of NME isolates from Greece and 1.3% of NME from Italy and accounted for most IMI/REL-resistant NME isolates from both countries (81%, 99/122) confirming results from an earlier study of this resistance phenotype [4]. Twelve of 56 NME isolates from Italy that carried an OXA-48-like enzyme (MBL-negative) were also IMI/REL-resistant.

Country-specific differences in percent susceptible values for C/T, IMI/REL, meropenem, piperacillin/tazobactam, levofloxacin, and amikacin were more pronounced for *P. aeruginosa* than Enterobacterales (Table 1). C/T and IMI/REL both inhibited 84% of all collected *P. aeruginosa* isolates from Greece and 91–92% of *P. aeruginosa* from Italy. Our results agree closely with those previously reported by Sader et al. for C/T tested against clinical isolates of *P. aeruginosa* collected between 2012 and 2018 in both Greece (81% susceptible; *n*=284) and Italy (93%; *n*=881) [7]. In the current study, MBL genes were identified in 10.2% of *P. aeruginosa* isolates from Greece compared to 2.7% of *P. aeruginosa* isolates from Italy (Table 2). GES carbapenemase genes were only identified in *P. aeruginosa* isolates from Italy (1.7% of isolates). MBL or GES genes were identified in 63% (17/27) of characterized C/T-resistant and 68% (17/25) of characterized IMI/REL-resistant *P. aeruginosa* isolates from Greece and 53% (36/68) of characterized C/T-resistant and 51% (37/73) of characterized IMI/REL-resistant *P. aeruginosa* isolates from Italy. Elevated PDC expression (in combination with OprD deficiency for IMI/REL) may account for some of the carbapenemase-negative C/T-resistant and IMI/REL-resistant isolates [19]. C/T inhibited 13.8% (4/29) of IMI/REL-resistant (MIC >2 mg/L) *P. aeruginosa*, and IMI/REL inhibited 16.7% (5/30) of C/T-resistant (MIC >4 mg/L) *P. aeruginosa* from Greece, compared with C/T inhibition of 30.8% (24/78) of IMI/REL-resistant *P. aeruginosa* and IMI/REL inhibition of 25.0% (18/72) of C/T-resistant *P. aeruginosa* from Italy.

Colistin (>99% susceptible) was the agent with the highest percent susceptible value for *P. aeruginosa* from both Greece and Italy, while only 71–81% of isolates were susceptible to meropenem, cefepime, ceftazidime, and piperacillin/tazobactam. Meropenem percent susceptible values for *P. aeruginosa* isolates from both Greece and Italy agreed with previous studies reporting carbapenem-resistant *P. aeruginosa* rates in southern and eastern Europe [4, 5]. Less than 50% of meropenem-resistant and other β-lactam-resistant isolate subsets of *P. aeruginosa* were levofloxacin-susceptible. C/T, IMI/REL, and amikacin also demonstrated limited activity (<40% susceptible) against meropenem-, cefepime, ceftazidime-, and piperacillin/tazobactam-resistant isolates from Greece. In contrast, against *P. aeruginosa* isolates from Italy, both C/T and IMI/REL retained activity against 70–74% of ceftazidime- and piperacillin/tazobactam-resistant isolates, while 53% and 36% of meropenem-resistant isolates were C/T-susceptible and IMI/REL-susceptible, respectively.

In considering the colistin and amikacin data, it is important to note that given the limitations associated with colistin and aminoglycoside use in treating gram-negative infections, EUCAST only publishes bracketed colistin, amikacin (systemic infections), and gentamicin (systemic infections) susceptible and resistant MIC breakpoints with a warning against the use of any of these agents without additional therapeutic measures [14, 20]. Similarly, CLSI does not publish a susceptible MIC breakpoint for colistin for any gram-negative pathogen [13].

In conclusion, despite noteworthy carbapenemase gene carriage among isolates of Enterobacterales and *P. aeruginosa* from both Greece and Italy, recent (2017–2021) clinical isolates of NME collected in Greece and Italy were highly susceptible (≥95%) to IMI/REL; susceptibility of Enterobacterales to C/T was approximately 10% lower (85–86%). *P. aeruginosa* collected in Greece were 7–8% less susceptible to both C/T and IMI/REL (84%) than isolates from Italy (91–92%) due to higher MBL carriage in isolates from that country. Geographic differences in β-lactamase/carbapenemase prevalence and other β-lactam resistance mechanisms are well established to affect the in vitro activities of currently available β-lactams and β-lactam/β-lactamase inhibitor combinations, including C/T and IMI/REL [2, 21, 22]. Based on our in vitro data, C/T and IMI/REL remain active against the majority of isolates Enterobacterales and *P. aeruginosa* from both Greece and Italy and may be important treatment options for patients in these two countries with infections caused by gram-negative pathogens.

## Data Availability

Data is available upon reasonable request.
